# Deregulation of Trace Amine-Associated Receptors (TAAR) Expression and Signaling Mode in Melanoma

**DOI:** 10.3390/biom12010114

**Published:** 2022-01-11

**Authors:** Anastasia N. Vaganova, Savelii R. Kuvarzin, Anastasia M. Sycheva, Raul R. Gainetdinov

**Affiliations:** 1 Institute of Translational Biomedicine, St. Petersburg State University, Universitetskaya nab. 7/9, 199034 St. Petersburg, Russia; anastasia.n.vaganova@gmail.com (A.N.V.); saveliy51@yandex.ru (S.R.K.); 2 Department of Pathology of Pavlov First Saint Petersburg State Medical University, Ministry of Health of Russia, Build. 30, L’va Tolstogo Str. 6–8, 197022 Saint Petersburg, Russia; ussanas@mail.ru; 3 St. Petersburg University Hospital, St. Petersburg State University, Universitetskaya nab. 7/9, 199034 St. Petersburg, Russia

**Keywords:** TAAR1, TAAR2, TAAR5, TAAR6, TAAR8, TAAR9, dopamine, melanoma, nevus, cancer

## Abstract

Trace amine-associated receptors (TAARs) interact with amine compounds called “trace amines” which are present in tissues at low concentrations. Recently, TAARs expression in neoplastic tumors was reported. In this study, TAARs expression was analyzed in public RNAseq datasets in nevi and melanoma samples and compared to the expression of dopamine receptors (DRDs) that are known to be involved in melanoma pathogenesis. It was found that all DRDs and TAARs are expressed in nevi at comparable levels. Differential expression analysis demonstrated the drastic decrease of TAAR1, TAAR2, TAAR5, TAAR6, and TAAR8 expression in melanomas compared to benign nevi with only TAAR6, TAAR8, and TAAR9 remaining detectable in malignant tumors. No association of TAARs expression levels and melanoma clinicopathological characteristics was observed. TAARs co-expressed genes in melanoma and nevi were selected by correlation values for comparative pathway enrichment analysis between malignant and benign neoplasia. It was found that coexpression of TAARs with genes inquired in neurotransmitter signaling is lost in melanoma, and tumor-specific association of TAAR6 expression with the mTOR pathway and inflammatory signaling is observed. It is not excluded that TAARs may have certain functions in melanoma pathogenesis, the significance of which to tumor progression is yet to be understood.

## 1. Introduction

Melanoma is a malignant neoplasm of the melanocytes, with development considered a consequence of an interaction between genetic susceptibility and environmental exposure [1]. Intensive ultraviolet (UV) irradiation with consequent skin injury and DNA damage is presumed the major risk factor for melanocyte malignization [2]. Other risk factors for melanoma are the presence of melanocytic nevi and some genetic variants, the being genes involved in cell cycle regulation [3,4]. Highly mutated melanoma becomes resistant to radiotherapy and cytotoxic agents; hence, the importance of revealing the specific therapeutic targets involved in melanoma genesis and progression.

The major pathways implicated in melanoma development and progression have also been found in other solid tumors. Around 40–60% of all melanomas exhibit a mutated BRAF proto-oncogene, which becomes continuously activated, stimulates the RAS-RAF-MEK-ERK kinase pathway, and promotes cell growth and proliferation. The activation of this pathway in melanoma also may be initiated by mutated NRAS GTPase or NF1 (neurofibromin) deficiency [4]. Uncontrolled signaling through the PI3K pathway is detected in 30–60% of melanomas [2,5,6]. Wnt signaling is critical for the development of melanocytes, but it is also involved in malignization [3]. UV-induced DNA damage causes TP53 missense mutations with high potential [7]. Copy number variations, translocations, copy gains, monosomy [8], epigenetic disturbances including CpG-islands hypermethylation and methylation loss in regulatory sequences, chromatin remodeling, the aberration in histone modification pattern, and deregulation of microRNA expression, are also described in melanomas [7]. Disruption of melanocyte-specific pathway regulation is also implicated in melanoma development. Reduction in the vitamin D receptor (VDR), melanocyte-stimulating hormone receptor (MC1R), and melanocyte-inducing transcription factor (MITF) expression is associated with tumorigenesis and disease progression [2,4,8]

Trace amines (TAs) have been described as a group of amines presented in mammalian tissues in low concentrations (1–100 ng. per g.), distributed within the brain in a heterogeneous fashion, present in a synaptosomal fraction after homogenization, and synthesized in vivo primarily by decarboxylation of their parent amino acids. These amines were named “trace” in contrast to the closely related classical monoamine transmitters, catecholamines, and 5-hydroxytryptamine [9]. Nowadays, this group includes both endogenous and exogenous molecules. Trace amine-associated receptors (TAARs) recognize both trace amines and some other amine compounds. In humans, TAAR1, TAAR2, TAAR5, TAAR6, TAAR8, and TAAR9 receptors are functional [10]. The expression of most TAARs (TAAR2-TAAR9) is found in the olfactory epithelium [11] but also in the limbic brain areas where the intended role of this receptor family is related to emotional behavior regulation in response to socially important or dangerous signals, and regulation of adult neurogenesis [12,13,14]. Nowadays, TAARs are detected in different organs and tissues, but the function of these receptors outside the nervous system remains elusive [15].

Recently, deregulation of TAAR1 receptor expression was revealed in sarcoma and esophageal, lung, stomach, cervical, renal, kidney, liver, pancreas, pituitary, prostate, urinary, and uterine cancers [16]. Mutated TAAR9 was identified as a tumor neo-antigen in murine melanoma cells [17]. TAAR1, TAAR2, TAAR5, TAAR6, TAAR8, or TAAR9 expression in breast tumors is positively associated with favorable survival outcomes [18]. Cadaverine binding to TAAR1, TAAR8, and TAAR9 modulates neoplastic properties of breast cancer cells [18], and TAAR1 expression is enabled in response to the growing concentration of its ligand T1AM in vitro [19]. TAAR1 is also a positive prognosticator in epithelial ovarian cancer [16]. Thus, a growing body of evidence suggests that the TAARs family may represent a novel target for cancer treatment and/or prognostic markers.

Some TAARs downstream signaling pathways involve heterodimerization with other monoaminergic G protein-coupled receptors (GPCR). The most studied of such interactions is TAAR1 heterodimerization with the DRD2 dopamine receptor [10]. Dopamine receptors (DRDs) are currently considered prospective therapeutic targets in oncology. DRD ligand impact on tumor growth may be mediated by cAMP/PI3K/AKT/CREB [20,21], ERK/MAPK [22], or Wnt/β-catenin signaling pathways [23]. The antipsychotic DRD2s antagonists thioridazine and fluphenazine induce differentiation of stem-like cancerous cells [24]. The effect of DRD ligands on neoplastic growth is context-dependent. DRD1 agonists reduce viability and promote apoptosis in triple-negative breast cancer cells but not in the luminal cell line MCF-7. Inhibition of DRD2 expression suppresses proliferation in breast cancer cell lines but promotes proliferation in non-small cell lung cancer cells [25]. DRD1 and DRD2 are expressed in melanoma samples and nondamaged human skin [26,27,28,29]. Dopamine leads to a marked cell proliferation decrease in melanoma cells [30]. It also is cytotoxic against noncancerous melanocytes [28,31]. DRD4 receptor antagonists reduced the melanin contents, MITF expression, and, consequently, ERK activation in melanoma cells [32].

Since current information about TAARs presence in melanoma is absent, we attempted to explore possible TAARs expression using public transcriptomic datasets in benign and malignant melanocytic neoplasm and analyzed the factors affecting the expression of TAARs genes in melanoma. This study aims to estimate TAARs expression in melanoma and the landscape of TAARs-related pathways in this malignant tumor. In the context of the aforementioned, we also reviewed DRDs expression in these tumors and compared the level of their expression to that of TAARs. Public “omics” data including transcriptomic datasets from the GEO database and Single Cell Portal were used as the data source for this study.

## 2. Materials and Methods

### 2.1. Data Collection and Inclusion Criteria for Datasets

Publicly available transcriptome datasets were retrieved from the GEO repository [33]. First, the GEO browser available from http://www.ncbi.nlm.nih.gov/geo/browse/ (accessed on 15 November 2021) was searched for the term “melanoma”. RNAseq-generated datasets containing data for the whole-tissue melanoma samples were analyzed.

Considering relatively low levels of GPCR expression [34,35], datasets with a small sample size were excluded to prevent sampling bias. The inclusion criteria were at least five samples from melanoma patients or other group included in the study. In view of the aim of the study, only datasets containing the data for TAARs expression were reviewed. Because of the weak TAARs mRNA transcription, its expression patterns may be estimated by RNAseq only if the appropriate sequencing depth is applied. To prevent overload with false-negative results, a mean read number in SRA files in the dataset of 50 million or higher was implemented as the threshold for dataset selection. After the exclusion of nonrelevant datasets, six RNAseq-generated datasets were selected for the analysis (Table 1, Table S1).

### 2.2. RNAseq-Generated Datasets Analysis

RNA sequencing datasets consist of row counts or FPKM-normalized data. So, for uniform estimation of the expression levels, all data were TPM-normalised. TPM values above the threshold level 0.5 were considered positive. The distribution of TPM-normalized expression levels in melanoma samples was visualized by the beeswarm R package.

### 2.3. Single-Cell RNA-Seq Data

For the single-cell RNA-seq data, the Single Cell Portal database (available on https://singlecell.broadinstitute.org/single_cell, accessed on 4 December 2021) was searched for the term “melanoma”. All datasets without data for TAARs expression were excluded, and the two remaining datasets were included in the review (Table 1). The receptor distribution in cell populations was analyzed and visualized by the Single Cell Portal interface.

### 2.4. Statistical Analyses

As the public data included in the review were represented in different units, two slightly distinct approaches were implemented for data normalization before differential expression analysis to ensure the accuracy of the result. To estimate the differential gene expression in datasets represented in row counts, the data were normalized using the Trimmed Mean of M-values (TMM) method by the edgeR package [36] to avoid batch effects. Then, the gene expression data were normalized by the voom function in limma [37]. If data were represented in fragments per kilobase million (FPKM), numeric values were log2-normalized (FPKM + 1 values were used for calculations).

After data prearrangement, differentially expressed genes were identified by the empirical Bayes test using the limma package [37]. Normalized data were fed to lmFit and eBayes functions. For log2 normalized RKPM, trend  =  TRUE in the eBayes function was applied. *p*-values for empirical Bayes moderated t-statistics were adjusted for multiple testing corrections using the Benjamini–Hochberg method. We considered differentially expressed genes in analysis with adjusted *p*-values (*P_adj_*) < 0.05.

Violinplotter free R package was applied for the result visualization.

### 2.5. Pathway Enrichment Analysis

In the GSE98394 dataset TAARs co-expressed gene clusters were selected by Pearson correlation coefficient as described in the results (*p* < 0.05). KEGG pathway enrichment analysis (identification of the known pathways that are significantly enriched by the genes of the cluster) in the selected clusters and visualization of results was performed by the clasterProfiler R Bioconductor package [38].

## 3. Results

### 3.1. TAARs and Dopamine Receptors Expression in Melanoma

**Figure 1:**
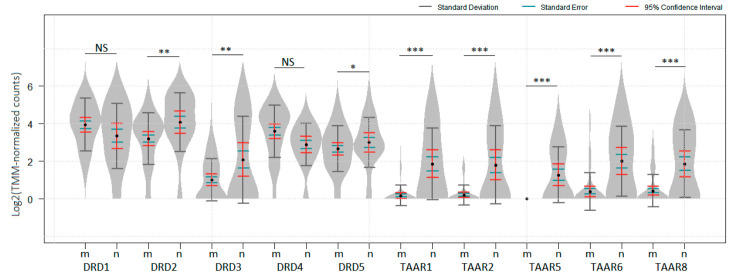
Expression levels of dopamine receptors and TAARs (no data available for TAAR9) in melanoma and nevi in GSE98394 dataset. Gene expression was compared in 51 melanoma (m) samples and 27 common acquired nevi (n) samples. *p* values were obtained from the empirical Bayes moderated t-statistic, * *P_adj_*  <  0.05, ** *P_adj_ * <  0.01, *** *P_adj_*  <  1 × 10^−5^, NS—not significant. The standard deviation, standard error and 95% confidence interval error bars are marked by gray, green and red colors respectively.

To evaluate TAARs and DRDs expression in benign and malignant melanocytic neoplasm, the dataset GSE98394, which comprised transcriptomic data for 27 common acquired nevi samples and 51 melanomas [39], was analyzed. All TAAR1, TAAR2, TAAR5, TAAR6, TAAR8, and all dopamine receptors, were identified in nevi samples included in this dataset. DRD2, DRD3, DRD5, and all identified TAARs were significantly downregulated in melanomas compared to nevi samples with the most drastic changes noted in TAARs (Figure 1, *p* < 0.05). No data for TAAR9 was represented in GSE98394.

**Figure 2:**
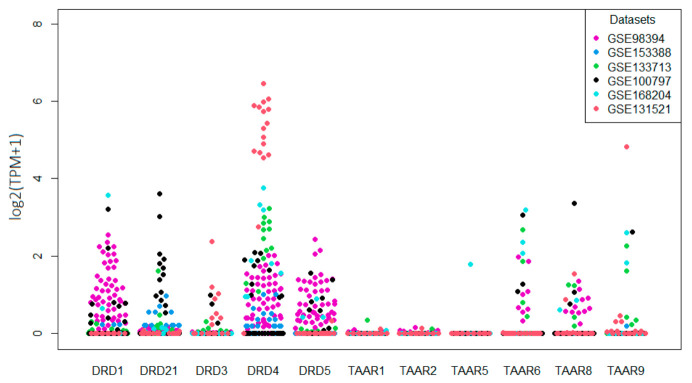
Expression levels of dopamine receptors and TAARs in melanoma samples in whole tissue samples are described in the GEO database.

Furthermore, the transcriptomic data for five additional RNAseq GEO datasets for 82 whole-tissue melanoma samples were included in the review. Following mined data, all dopamine receptors were detected in melanomas; among them DRD1 in 33.8%, DRD2 in 11.3%, DRD3 in 4.5%, DRD4 in 62.4%, and DRD5 24.1% of samples (Figure 2). TAARs expression was pronouncedly lower than that of dopamine receptors. No samples expressed TAAR1 and TAAR2 mRNA at levels over the cut-off 0.5 TPM, and only one melanoma sample was positive for TAAR5 expression. In contrast, TAAR6, TAAR8, and TAAR9 were expressed in 12.7%, 11.3%, and 5.3% of tumor specimens, respectively (Figure 2), so their expression is comparable with DRD2, of which expression and activity are well established in melanoma [27,40].

All receptors, except DRD4, are expressed at homogeneous levels in the different datasets. The variations may be attributed to the batch fluctuations and technological differences. Specifically, high DRD4 expression in tumor samples of the GSE131521 dataset could be associated with the particular properties of these samples because all of them were collected from patients with metastatic brain damages [41].

### 3.2. Loss of TAARs and Dopamine Receptors during Tumor Progression

**Figure 3:**
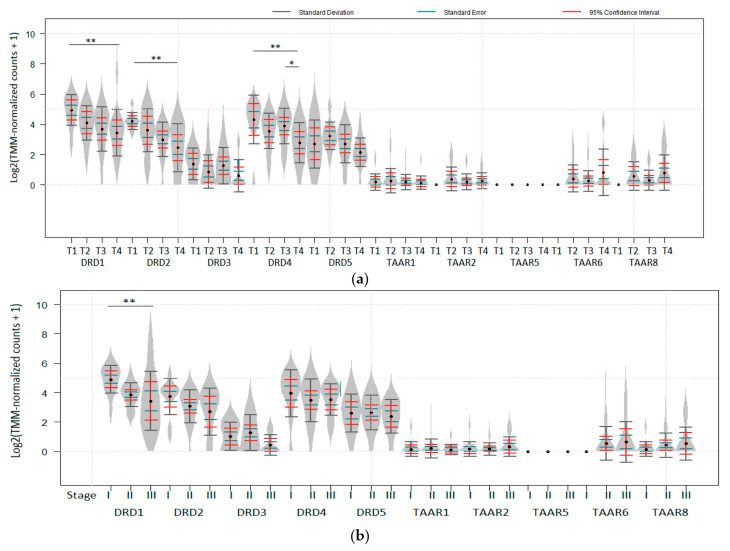
Deregulation of dopamine receptors expression during melanoma progression (RANseq data). (**a**) DRD1, DRD2, and DRD4 expression levels in melanoma are associated with tumor thickness (T stage) in GSE98394 dataset (n (T1) = 10, n (T2) = 10, n (T3) = 10, n (T4) = 10). (**b**) Downregulation of DRD1 in melanoma is associated with cancer stage progression in GSE98394 dataset (n (Stage I) = 12, n (Stage II) = 22, n (Stage III) = 10). * *P_adj_*  <  0.05, ** *P_adj_*_ _ <  0.01. No data for TAAR9 expression available in the dataset. *p* values were obtained from the empirical Bayes moderated t-statistic, * *P_adj_ * <  0.05, ** *P_adj_*  <  0.01. Standard deviation, standard error and 95% confidence interval error bars are marked by gray, green and red colors respectively.

The following data were available for datasets included in the review: the impact of tumor thickness (T stage); involvement of lymph nodes (N stage); distant metastases (M stage), and localization (subcutaneous vs. lymph nodes for metastatic lesions) on the expression of dopamine receptors and TAARs. In RNA-seq generated dataset GSE98394, DRD1, DRD2, and DRD4 expression was significantly lower in T4 than in T1 tumors (Figure 3a). When American Joint Committee on Cancer (AJCC) staging was considered, DRD1 was also significantly higher expressed in Stage I tumors than in more progressed melanoma (*P_ad_*_j_ < 0.05, Figure 3b). Neither dopamine receptors DRD3 and DRD5, nor TAARs were significantly downregulated in late-stage tumors in this dataset. No impact of N and M stages or metastases localization was revealed in the datasets included in the review.

**Figure 4:**
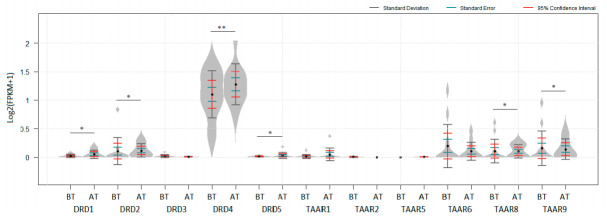
Deregulation of dopamine receptors and TAARs after hu14.18-IL2 treatment in GSE133713: BT—before treatment n = 13, AT—after treatment, n = 10. * *P_adj_ * <  0.05, ** *P_adj_*  <  1 × 10^−5^. No data for TAAR9 is represented in the dataset. *p* values were obtained from the empirical Bayes moderated t-statistic: * *P_adj_ * <  0.05. ** *P_adj_*  <  1 × 10^−8^. Standard deviation, standard error and 95% confidence interval error bars are marked by gray, green and red colors respectively.

The dataset GSE133713 represents the transcriptomic data for tumor samples that were resected in the course of clinical trial CO05601. Patients with Stage III–IV melanoma were included in the study and randomized to have their first of three monthly courses of hu14.18-IL2 started either just before (Group A, n = 13) or just after (Group B, n = 8) their complete surgical resection [42]. Significant up-regulation of DRD4 expression in melanomas after hu14.18-IL2 treatment was found (Figure 4, *P_adj_* < 1 × 10^−8^). TAAR8, TAAR9, DRD1, DRD2, and DRD5 expression was also slightly upregulated in melanomas after immunotherapy (Figure 4, *P_adj_* < 0.05). Likely because of differences in statistical approaches, no significant variations of DRDs expression levels were reported in the original paper [42].

### 3.3. Distribution of TAARs and Dopamine Receptors mRNA between Cell Populations in Melanoma

**Figure 5:**
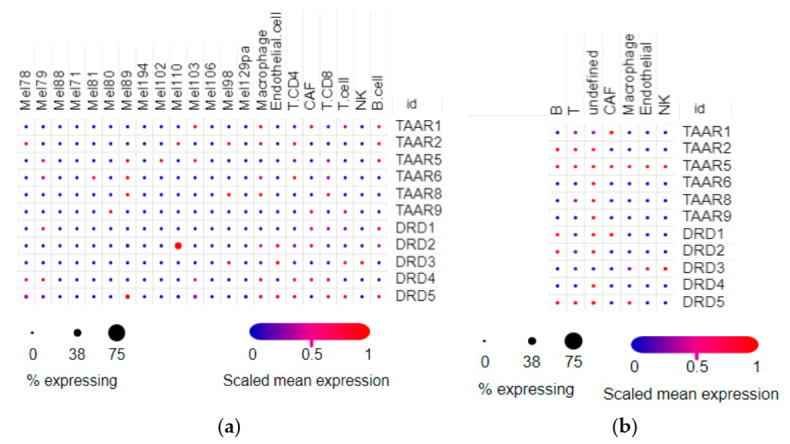
Distribution of dopamine receptors and TAARs expression in cell populations in melanoma samples. (**a**) Expression profiles in different subtypes of melanoma cells and stromal cells in the SCP109 dataset. (**b**) Expression profiles in stromal cells in the SCP11 dataset (pictures were generated by the Single Cell Portal interactive interface).

The cell composition of tumor samples is heterogeneous, so two single-cell RNAseq datasets were included in the review. In the SCP109 dataset (Figure 5a), tumor cells sporadically expressed all TAARs and DRDs. Only accidental cases of these gene expressions were detected in different tumor cell groups, and only the DRD2 receptor was pronouncedly higher expressed in one population of tumor cells (Mel 110 malignant cells in accordance with cell naming in the dataset). In the stromal cells, TAARs and DRDs were expressed sporadically in cells involved in the innate or specific immune response. TAAR1 and TAAR9 were also detected in cancer-associated fibroblasts (CAF) (Figure 5a).

Under the described expression pattern in the SCP11 dataset, TAARs were expressed at low levels in accidental cells. TAAR1 was expressed in CAF, and other TAARs were expressed in unidentified cells. The expression levels of dopamine receptors were comparable with TAARs (Figure 5b).

### 3.4. Deregulation of TAARs-Related Biological Processes in Melanoma

The correlation between expression of TAARs and other genes in the melanoma transcriptome considerably declined compared to the TAARs coexpression pattern in nevi in dataset GSE98394. In common nevi 3521, 3556, 2961, 3562, and 3247 genes expression significantly correlated with TAAR1, TAAR2, TAAR5, TAAR6 and TAAR8 expression levels, respectively, (r > 0.7, *p* < 0.05). In contrast, in melanoma, only 34, 73, 6, 137 and 146 genes’ expression correlated with the expression levels of TAAR1, TAAR2, TAAR5, TAAR6, and TAAR8 (r > 0.7, *p* < 0.05), considering that TAAR1, TAAR2, and TAAR5 expression levels in melanoma were below the cut-off value 0.5 TPM. As the TAARs coexpressed gene clusters in nevi are considerably greater than TAARs coexpressed gene clusters in melanoma, only the genes whose expressions correlated with the appropriate TAARs at the level r > 0.9 (*p* < 0.05) were included in pathway enrichment analysis in the nevi.Pathway enrichment analysis, which showed no KEGG pathway enrichment in the TAAR8 coexpressed gene cluster in melanoma and the TAAR5 co-expressed cluster in nevi.

**Figure 6:**
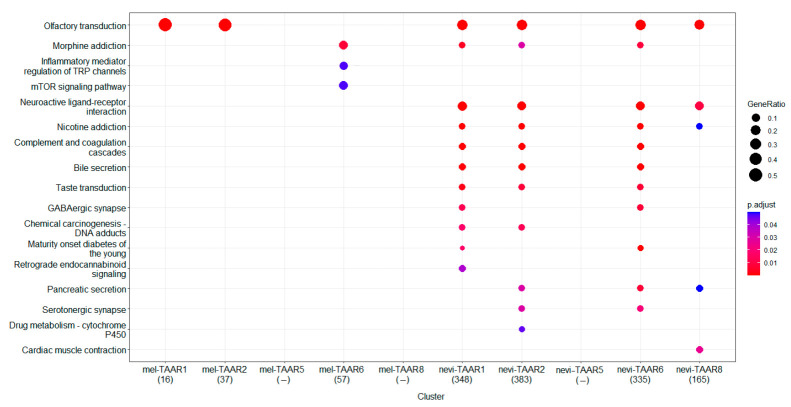
KEGG pathways enriched by genes coexpressed with TAARs (GSE98394, no data for TAAR9 expression is available) in melanoma and nevi. mel-TAAR6—TAAR6 coexpressed gene cluster in melanoma, nevi-TAAR2, nevi-TAAR6, nevi-TAAR8—TAAR2, TAAR6, or TAAR8 coexpressed gene cluster in nevi, respectively. Point colors indicates the Benjamini-Hochberg adjusted *p*-values for Fisher’s exact test.

Despite the number of genes in the TAAR6 coexpressed cluster in melanoma being slightly less than in the TAAR8 coexpressed cluster, this gene set is enriched in three KEGG pathways (Figure 6). The pathway “Morphine addiction” is the most enriched by genes of this cluster which was also found in TAAR1, TAAR2, or TAAR6 coexpressed gene clusters in nevi. This comprises genes that encode components of GABA-ergic synapses on ventral tegmental area dopaminergic neurons. The enrichment analysis confirms loss of TAAR6 association with other pathways in which TAARs associated genes are enriched in nevi (Figure 6). Instead, the TAAR6 coexpressed gene cluster in melanoma is enriched in pathways “Inflammatory mediator regulation of TRP channels” representing processes in nociceptive dorsal root ganglion (DRG) neurons, and “mTOR signaling pathway”.

Other relations with neurotransmitter-signaling and GPCR pathways were revealed in nevi, including pathways “Olfactory transduction”, “Neuroactive ligand-receptor interactions”, “Nicotine addiction”, “Taste transduction”, “GABA-ergic synapse”, “Pancreatic secretion”, and “Serotoninergic synapse”. These pathways were entirely lost in TAARs coexpressed clusters in melanoma. It is notable that, while TAAR1 and TAAR2 expression in melanoma was below the cut-off value, genes involved in the “Olfactory transduction pathway,” coupled with these receptors in nevi were retained in melanoma samples (Figure 6).

This analysis revealed the loss of normal TAARs function in melanoma and the potential involvement of TAAR6 in some melanoma-specific processes. Only 16 genes were common for TAAR6 co-expressed clusters in melanoma and nevi, and all these genes were involved in the “Morphine addiction” pathway (i.e., genes coding GABA receptor subunits gamma2 and rho1).

## 4. Discussion

The encouraging therapeutic potential of TAAR1 agonists as the first antipsychotic drugs without DRD2 blocking action to treat schizophrenia has been demonstrated in clinical trials [43]. Nevertheless, with the accumulation of knowledge about TAARs significance outside the nervous system, it becomes clear that the range of diseases that may be treatable by TAARs ligands may be broader and include, for example, immunomodulatory diseases [44]. As the association between TAARs expression and oncologic diseases prognosis has been described [16], this study aimed to uncover possible TAARs-related biological process impairment in melanoma.

Earlier, most TAARs (TAAR2-TAAR9) were considered exclusively as olfactory receptors [11], but there is growing evidence that they play an important role in different brain areas, neurogenesis, as well as in functioning of the immune, endocrine, and digestive systems [10,12,13,45]. TAARs function in non-neural cells seems to be a promising topic for further research because they can be the possible targets for new drug development for various disorders.

Melanocytes originate from neural crest cells, having a common progenitor with Schwann cells, and share some characteristics with neurons where TAARs functions have been intensively investigated. Melanocytes have some morphological or molecular similarities with neuronal cells, such as some features of signal transmission, dendrite formation, spine formation, and directional organelle transport [46,47]. In addition, melanin synthesis is characteristic of both dopamine neurons and melanocytes and includes L-DOPA as a precursor.

In this study, TAAR6, TAAR8, and TAAR9 expression was found in melanoma samples. TAAR1, TAAR2, and TAAR5 were expressed at extremely low values that did not reach cut-off 0.5 TPM, so their expression was considered as negative. As previously demonstrated, dopamine receptors are also expressed in melanomas. Public data analysis revealed that TAAR6 and TAAR8 expression levels are low (i.e., below 10 TPM) but comparable with DRD2. As previously described, DRD2 is active in melanoma and, despite its low expression, can bind specific ligands in detectable levels [26] and mediate the antineoplastic effect of DRD2 blocking antipsychotics like phenothiazines [48,49]. Phenothiazines induce apoptosis in a B16 mouse melanoma cell line and attenuate in vivo melanoma tumor growth or the small DRD2/3 binding molecule ONC201, which induces anticancer effect mediated by activation of the death ligand TRAIL in tumor [29,50]. As TAAR1 is coexpressed and heterodimerizes with DRD2, and can manifest its physiological effects in the brain despite its low presence [10], these receptors may also be prospective therapeutic targets in melanoma. Single-cell RNAseq datasets demonstrate that TAARs and dopamine receptors are expressed in both tumor and stromal cells at low levels.

Dopamine receptors and TAARs mRNA levels are downregulated in melanoma compared to benign nevi in the GSE98394 dataset. Previously, dramatic differences between melanoma and nevi gene expression patterns were found in these transcriptomic data. At least 4.396 genes were differentially expressed between benign and tumor samples, including genes regulating immune response and inflammation, cell cycle and proliferation, epigenetic modifiers, apoptosis, extracellular matrix, and cell adhesion [39].

Some slight associations between DRDs expression levels and tumor progression were observed. DRD1, DRD2, and DRD4 expression was lower in T4 tumors compared to less thick tumors. DRD1 expression was also significantly lower in Stage III tumors than in Stage I tumors. No other association with TAARs or DRDs and clinicopathological characteristics were revealed in this study.

The enrichment analysis revealed the disregulation of TAARs associated pathways in melanoma. The TAARs coexpressed gene numbers in tumor samples were dramatically lower than in benign nevi. In nevi, TAARs expression seems to be associated with the plethora of genes involved in GPCR-dependent biologic processes, neurotransmitter signaling, and external chemical stimuli sensing. Interestingly, the pathway mainly enriched with the TAARs coexpressed gene is the “Morphine addiction” KEGG pathway, which involves perturbations in GABA and dopamine signaling in the ventral tegmental area. Thus, TAARs expression in benign melanocytes seems to be involved in recognizing neurotransmitters or some other signals in cutaneous circumstances.

There are several limitations of the present study. The majority of transcriptomic data which were included in the review characterize late-stage disease and metastatic lesions, but the data for early-stage melanoma are limited. The transcriptomic datasets generated with appropriate sequencing depth for uveal melanoma are also unavailable, and only cutaneous lesions were analyzed. The patients and disease characteristics in datasets were also incomplete, often lacking the data about sex/age in the population, and molecular tumor profiles, such as BRAF, NRAS, or p53 mutation status were completely lost. Bearing in mind all these limitations, it is important to note that the expression levels of TAARs and DRDs in different datasets seem to be broadly similar. As the pathway enrichment analysis was performed on whole tissue RNAseq data, it was impossible to distinguish gene expression in malignant melanocytes and stromal cells. Pathway enrichment results did not clearly indicate the nature of the association of TAARs with identified biological processes, and the results were insufficient to determine if TAAR6 was implicated in cancer-associated pathways, or TAAR6 only co-regulates with mTOR signaling in tumors.

Keratinocytes and melanocytes produce neurotransmitters, including catecholamines [51,52]. Skin also is intensively innervated by noradrenergic and cholinergic nerves. Catecholamines seem to participate in intercellular interaction in skin and regulation of melanin production and, possibly, melanocyte survival [28]. The presence of TAs which are closely related to catecholamines in melanocytes and the inclusion of these compounds is biological processes in the skin remain uncharacterized. The TAARs function in melanocytes is unknown, but the present study observed substantial deregulation of TAARs in melanomas compared to benign nevi. These receptors may be involved in melanocyte sensing endogenous and exogenous amine compounds that should be identified in future studies. The results indicate that deregulation of both dopamine and trace amine signaling may complement the malignization of melanocytes.

## 5. Conclusions

Public data provides opportunities for systemic analysis, including the coverage of questions that were not raised when the data were obtained. In this study, we collected and reviewed datasets to mine new data about the expression of TAARs and functionally adjoint dopamine receptors in melanoma. Differential gene expression analysis revealed a prominent decrease in TAARs expression in malignant melanoma compared to benign melanocytic nevi where all TAARs were expressed at levels comparable to dopamine receptors. Essentially undetectable TAAR1, TAAR2, and TAAR5 expression levels were observed in melanoma samples. However, TAAR6, TAAR8, and TAAR9 were still expressed in melanoma. The downregulation of TAARs expression in tumor tissue was accompanied by the loss of their involvement in cell regulation and receptor activity. At the same time, the acquisition by TAARs, especially by TAAR6, of any new functions specific to the malignant state could not be excluded. Further studies are needed to explore if TAARs can serve as novel targets for the treatment of melanoma, and to evaluate their potential prognostic value.

## Figures and Tables

**Table 1:** RNAseq datasets included in the review.

Dataset ID	Title	n ^1^	Samples Characteristics	Sex	Age Range (Years)
Whole tissue					
GSE98394	Transcriptional dissection of melanoma identifies a high-risk subtype underlying TP53 family genes and epigenome deregulation	51 ^2^	Stage I–IV melanoma	31 male/20 female	Not specified
GSE100797	Mutational and neoantigen load predict clinical benefit of adoptive T cell therapy in melanoma	25	Stage IV melanoma	Not specified	Not specified
GSE131521	Radiation enhances melanoma response to immunotherapeutic and synergizes with benzodiazepines to promote improved anti-tumor activity	17	Brain metastases	13 male/4 female	34–81
GSE133713	Immune signatures and tumor biomarkers from whole transcriptome sequencing predict outcome in recurrent resectable stage III and IV melanoma when evaluated following treatment with Hu14.18-IL2	23	13 pretreatment and 10 post treatment	15 male/8 female	21–69
GSE153388	Inflammatory transcript signatures and activated regulatory T lymphocytes in melanoma met	19	Cutaneous metastases	Not specified	29–83
GSE168204	Pathway signatures derived from on-treatment tumor specimens predict response to anti-PD1 blockade in metastatic melanoma	20	8 pretreatment and 12 on-treatment samples	Not specified	21–70
Single cells (scRNAseq data)					
GSE115978/SCP109 ^3^	Single-cell RNA-seq of melanoma ecosystems reveals sources of T cells exclusion linked to immunotherapy clinical outcomes	7186 cells	31 tumor specimens	21 male/10 female	37–83
SCP11	Melanoma intra-tumor heterogeneity	2478 cells	19 tumor specimens	12 male/7 female	43–86

^1^ The number of sequenced melanoma samples in the dataset is mentioned. ^2^ The dataset also includes 27 common acquired neve samples. ^3^ Dataset IDS in GEO and Single Cell Portal respectively.

## Data Availability

All whole tissue sample data are available in the GEO database (https://www.ncbi.nlm.nih.gov/geo/ (accessed on 10 December 2021), the detailed information is listed in Table S1). Single-cell RNAseqdatasets included in the review are available online at Single-CellPortal (https://singlecell.broadinstitute.org/single_cell (accessed on 10 December 2021), i.e., Study: Melanoma intra-tumor heterogeneity—https://singlecell.broadinstitute.org/single_cell/study/SCP11/melanoma-intra-tumor-heterogeneity (accessed on 10 December 2021); Study: Melanoma immunotherapy resistance—https://singlecell.broadinstitute.org/single_cell/study/SCP109/melanoma-immunotherapy-resistance (accessed on 10 December 2021).

## Supplementary Materials

The following supporting information can be downloaded at: https://www.mdpi.com/article/10.3390/biom12010114/s1. Table S1: GEO datasets included in the review.
