# Supplementary material for: Deregulation of Trace Amine-Associated Receptors (TAAR) Expression and Signaling Mode in Melanoma
Source: Biomolecules. 2022 Jan 11;12(1):114. doi: 10.3390/biom12010114 (PMC8774021; doi:10.3390/biom12010114)
Supplement: Supplementary file 1 [file biomolecules-12-00114-s001.zip › biomolecules-1545024-supplementary.pdf]

Table S1: GEO datasets included in the review.

GSE identifier	Authors	Title	Structure	Platform	Link
GSE100797	Lauss M, Jönsson G	Mutational and neoantigen load predict clinical benefit of adoptive T cell therapy in melanoma	25 melanoma samples	Illumina HiSeq 2000	https://www.ncbi.nlm.nih.gov/geo/query/acc.cgi?acc=GSE100797
GSE131521	Xu M, Sengupta S, Krummel D, Khan M	Radiation enhances melanoma response to immunotherapeutic and synergizes with benzodiazepines to promote improved anti-tumor activity	17 brain metastases	Illumina HiSeq 2500	https://www.ncbi.nlm.nih.gov/geo/query/acc.cgi?acc=GSE131521
GSE133713	Khan J, Wei JS	Immune Signatures and Tumor Biomarkers from Whole Transcriptome Sequencing Predict Outcome in Recurrent Resectable Stage III and IV Melanoma when Evaluated Following Treatment with Hu14.18-IL2	13 pre-treatment and 10 post-treatment samples	Illumina NextSeq 500	https://www.ncbi.nlm.nih.gov/geo/query/acc.cgi?acc=GSE133713
GSE153388	van Baren N, Mercier M, de Streel G	Inflammatory transcript signatures and activated regulatory T lymphocytes in melanoma metastases	19 cutaneous metastases	Illumina HiSeq 2000	https://www.ncbi.nlm.nih.gov/geo/query/acc.cgi?acc=GSE153388
GSE168204	Gao Z, Tian T	Pathway Signatures Derived from On-treatment Tumor Specimens Predict Response to Anti-PD1 Blockade in Metastatic Melanoma	27 melanoma samples	Illumina HiSeq 2000 / Illumina NextSeq 500	https://www.ncbi.nlm.nih.gov/geo/query/acc.cgi?acc=GSE168204
GSE98394	Badal B, Solovyov A, Di Cecilia S, Chan JM, Chang L et al	Transcriptional dissection of melanoma identifies a high-risk subtype underlying TP53 family genes and epigenome deregulation	27 benign melanocytic lesions and 51 melanoma samples	Illumina HiSeq 2500	https://www.ncbi.nlm.nih.gov/geo/query/acc.cgi?acc=GSE98394
GSE100797	Lauss M, Jönsson G	Mutational and neoantigen load predict clinical benefit of adoptive T cell therapy in melanoma	25 melanoma samples	Illumina HiSeq 2000	https://www.ncbi.nlm.nih.gov/geo/query/acc.cgi?acc=GSE100797
